# Competition Law and Ethics of Innovation as Catalysts for Fairness: Reimagining the EU’s COVID-19 Vaccine Strategy

**DOI:** 10.1017/jme.2025.10141

**Published:** 2025

**Authors:** Mina Hosseini

**Affiliations:** Sutherland School of Law, University College Dublin, Ireland

**Keywords:** Pharmaceutical Procurement, EU Vaccines Strategy, Advance Purchase Agreements, Competition Law, Ethics of Innovation

## Abstract

The “EU Vaccines Strategy” launched by the European Commission in June 2020 aimed to ensure vaccine safety, equitable access, affordability, swift distribution, and global solidarity for COVID-19 vaccines. This study critiques the Commission’s centralized procurement approach, focusing on Advance Purchase Agreements (APAs) through a literature review, policy analysis, and a case study of the EU-AstraZeneca’s APA. It identifies critical challenges, including transparency deficits, accountability gaps, and anticompetitive practices by vaccine producers that undermine equitable access. Drawing on these insights, the study proposes the FACER Framework — Fairness, Accountability, Competition Law, Ethics of Innovation, and Resilience — a novel model integrating the Treaty on the European Union (TEU) and the Treaty on the Functioning of the European Union (TFEU) oversight with ethical principles. By embedding legal and moral accountability, FACER offers EU policymakers a robust tool to enhance vaccine strategy and equity in future health crises.

## Introduction

In a reality where vaccine development typically spans a decade or more,^1^ the unprecedented acceleration to deliver safe and effective solutions for COVID-19 within a year resulted from enormous government support and funding, global cooperation, and coordination between the public and private sectors.^2^ New techniques in development and manufacturing — such as mRNA technology, designing structure-based antigens, and gene synthesis — allowed for the production of vaccines quickly and effectively in less than a year.^3^ EU policies towards vaccine procurement entailed relaxing specific regulations concerning genetically modified organisms,^4^ and labeling and packaging.^5^ During the pandemic, the European Commission adopted a pragmatic and flexible approach,^6^ utilizing soft laws including comfort letters^7^ to promote cooperation among undertakings, especially in developing vaccines and other countermeasures.^8^ These policies were implemented to facilitate vaccine development, manufacturing, and procurement, and they helped EU citizens and residents benefit from the result of such a flexible approach while also benefiting pharmaceutical companies and vaccine producers by increasing their revenue.

The global impact of the pandemic has emphasized the importance of pharmaceutical procurement in addressing public health crises. For all governments around the globe, rapidly developing and distributing COVID-19 vaccines has become the top priority. The EU’s vaccine plans have drawn much attention in this context. The EU vaccine strategy aims to guarantee the vaccine’s efficacy, safety, and quality, enable affordable vaccine access for all EU member states, and promote equal access to vaccines as part of the EU’s global solidarity plan.^9^

The legal format of the Commission’s vaccine procurement agreements with the COVID-19 vaccine producers was the “Advance Purchase Agreement” (APA).^10^ As an essential part of the EU’s vaccine strategy, APAs enabled the Commission to negotiate and purchase COVID-19 vaccines “on behalf and in the name of Member States.”^11^ This centralized strategy aimed to guarantee fair vaccine access while avoiding Member States’ competition on vaccine procurement,^12^ which could reduce supplies and raise prices. This study first sets the EU vaccine strategy in context, mapping its origins and implementation, and reviews the strengths and tensions of its APAs through the EU-AstraZeneca case study. It then explores how integrating competition law and ethics of innovation can enhance transparency and equity in vaccine procurement. Using these insights, the paper introduces the FACER Framework — Fairness, Accountability, Competition Law, Ethics of Innovation, and Resilience — a model that merges Treaty on European Union (TEU) and Treaty on the Functioning of the European Union (TFEU)-based legal oversight (including Article 102) with ethical principles to ensure equitable vaccine access. By adding these catalysts to the vaccine strategy, the study reveals how the EU can strengthen its vaccine strategy and response to health emergencies.

## The EU Vaccines Strategy: Context and Critique

The EU has been developing strategies and policies to promote vaccine development since long before the COVID-19 pandemic. The literature indicates that the EU had been advancing vaccine research and development to commercialize new and improved vaccines for years. The EU’s vaccine initiatives to facilitate collaboration between European industry, public institutions, and academia to support joint research initiatives have a long history, and vaccine research has benefited from the EU’s Framework Programmes^13^ and EU strategies to help promising vaccine candidates reach the market.^14^

Launched on June 17, 2020, by the European Commission, the EU Vaccines Strategy aimed to facilitate the creation, production, and rollout of safe and effective COVID-19 vaccines. The Commission negotiated the APAs with pharmaceutical companies, ensuring access to over 4.2 billion doses by committing funds from the €2.7 billion Emergency Support Instrument as an initial investment.^15^ In addition to prioritizing equitable access, the strategy supported global efforts through initiatives like COVID-19 Vaccines Global Access (COVAX), for which over 530 million vaccine doses were donated by August 2023. With the rise of new variants of COVID-19, the EU encouraged the refinement of its objectives and infrastructure, establishing the Health Emergency Preparedness and Response Authority (HERA) in late 2021.^16^

In 2022 and 2023, the Commission authorized adapted vaccines targeting Omicron subvariants, such as the BioNTech-Pfizer Comirnaty XBB.1.5, approved on August 31, 2023. National vaccination plans were encouraged to incorporate these updates, focusing on logistical readiness and prioritizing vulnerable groups. By August 2023, 84.8 percent of EU adults had received at least one dose, rising from 20 million doses monthly in early 2021 to 300 million by the middle of that year.^17^

In response to the need for greater preparedness against future health crises, as underscored by the COVID-19 pandemic and supported by the EU4Health program,^18^ the EU established the EU FAB network on June 30, 2023, contracting four producers in Belgium, Ireland, the Netherlands, and Spain to maintain flexible manufacturing capabilities for mRNA, vector-based, and protein-based vaccines, ready to deliver up to 325 million doses annually if a public health emergency arises.^19^ The European Commission adopted a centralized strategy for negotiating and buying COVID-19 vaccines on behalf of EU member states.^20^ The primary objective of this approach was to secure equitable and simultaneous access to vaccines for all EU countries while minimizing costs to the lowest possible level. By September 2020, the Commission had allocated €400 million to COVAX and joined international initiatives like Friends of COVAX to promote multilateralism and ensure equitable vaccine access.^21^ However, ethical tensions emerged: the centralized approach prioritized EU citizens, potentially exacerbating global inequities.^22^ Furthermore, as Sun and Sunder argued, the distribution of vaccines, unlike the development, was not a success story.^23^

The negotiation framework for purchasing COVID-19 vaccines included the establishment of a “Steering Board,” which consisted of one representative per Member State. The Commission and a Member State representative co-chaired this board. The European Court of Auditors (ECA) report (2022) found that the selection process for board members did not consider their knowledge and expertise.^24^ For the negotiation of purchasing COVID-19 vaccines, a Joint Negotiation Team (JNT) was established, and the Commission officials from various Director Generals (DGs) and representatives from the seven Member States of different subgroups of the JNT negotiated with a specific candidate vaccine manufacturer.^25^ After a market study (including sending questionnaires to candidates or having meetings with them), preliminary negotiations, getting approval from the Steering Board, and submitting a tender application by the candidate, the final negotiations would be followed by the contract signature.^26^ However, some negotiations diverged from the outlined framework, particularly with Pfizer/BioNTech (for over 900 million doses). In this case, the Commission engaged directly with pharmaceutical companies without involving the JNT.

### Centralized Vaccine Procurement

Before the COVID-19 pandemic, there were different methods of vaccine procurement, such as “self-procurement by states,” “donations by international organizations,” and “procurement and administration of vaccines by international organizations” to secure and supply vaccines.^27^ APAs facilitate vaccine development through legal agreements between manufacturers, governments, and international organizations. Payments are typically made during the development phase to expedite the manufacturing process. The use of APAs for vaccine procurement did not start with the COVID-19 pandemic. APAs were used to ensure vaccine availability during pandemics and epidemics pre-COVID-19. During the H1N1 influenza pandemic and outbreaks of Ebola and Zika, APAs were commonly used.^28^ As Turner discussed in his study, in the 2009 H1N1 pandemic, several states (including some EU Member States) used APAs to supply pandemic vaccines. Turner also discussed how countries after the 2009 H1N1 pandemic paid an annual “pandemic preparedness fee” to maintain the APA for vaccines in case of future pandemics.^29^

The Commission could secure advantageous prices and supply agreements for the Member States by centralizing vaccine procurement. Negotiating collectively led to an increase in bargaining power and a decrease in costs when compared to individual state negotiations. Investing in various vaccine options and having a portfolio of different kinds of COVID-19 vaccines helped the EU reduce the risk of shortages in case vaccine candidates failed or encountered production issues.^30^ Encouraging collaboration and cooperation between pharmaceutical firms and issuing comfort letters to facilitate the vaccine development process, research and development, and production of vaccines accelerated COVID-19 vaccine development and production.^31^ However, although the centralized system helped the EU procure a substantial vaccination supply, there were some drawbacks. According to Della Corte, the EU’s response to the COVID-19 crisis and the EU Vaccines Strategy may have failed to maximize the potential benefits to EU public health, and a joint approach left democratic and social participation out of its provisions.^32^ The EU was slower than the US and the UK in securing APAs. In the months following the development of COVID-19, vaccine shortages led to a slower vaccination process.^33^ The rollout of vaccines was significantly disrupted due to production and delivery issues by vaccine manufacturers. The EU had limited contingency plans available to address these challenges.^34^ The bureaucratic decision-making process, slow regulatory approval, and coordination difficulty between 27 countries were some of the reasons for the vaccine delay in the EU.

Despite the EU’s vaccine strategy focusing on joint procurement and promoting a centralized approach, each Member State followed a different vaccination policy. As Van Kessel et al. argued,^35^ a lack of alignment between internal health crisis policies of Member States could harm public health policy implementation and lead to lower public trust in this regard. Some Member States also followed parallel routes in vaccine procurement. For example, Germany signed separate agreements with vaccine producers. This raised serious doubts about the effectiveness of the centralized approach and the absence of sanctions for violating the agreement by Member States.^36^

Another EU policy, which is designed in line with the EU’s vaccine strategy, is the Pharmaceutical Strategy for Europe, which addresses broader concerns in the pharmaceutical sector and provides a framework for future health emergencies. Increasing accessibility of patients to “innovative and affordable drugs” and promoting the EU’s “pharmaceutical industry’s competitiveness, innovative capacity, and sustainability” are among the objectives of the “Pharmaceutical Strategy for Europe” adopted by the Commission on November 25, 2020.^37^ This policy can assist in ensuring that the EU is better prepared for future pandemics by increasing competition and innovation in this sector.^38^

Transparency and accountability are the factors that impact public trust.^39^ One of the concerns mentioned in the European Court of Auditors report (2022) was the lack of transparency in the COVID-19 vaccine negotiation process between the Commission and vaccine producers. As the World Health Organization (WHO) highlighted in its annual vaccine report, even delivery dates and allocation amounts were unclear in some vaccine procurement agreements.^40^

Another concern about APAs is that adopting these model agreements aggravates global vaccine inequities. As Phelan et al. emphasized, APAs enable early and priority access to vaccine procurement for wealthy countries that successfully negotiate with vaccine manufacturers. This disadvantages other countries facing financial or political barriers and could increase casualties — particularly in the Global South — as vaccine supply during a global health crisis remains limited.^41^

Vaccine producers used public money to develop and produce fast-track vaccines during a global pandemic, and their lack of accountability must be considered a legal risk in the next health crisis. For example, Pfizer’s CEO “not attending the European Parliament sessions to address the concerns of the European Ombudsman’s inquiry” can be considered a “lack of accountability” from the citizens’ viewpoint.^42^

### Analyzing the EU and AstraZeneca APA as a Case Study

The APA between the Commission and AstraZeneca (AZ) offers a compelling case study for analyzing various dimensions of such contracts. This example was chosen due to the multiplicity of disputes between the EU and AZ, such as disputes concerning delivery failures, opaque pricing, and liability provisions. This case vividly highlights the interplay between law and the ethics of innovation within the EU’s centralized vaccine procurement strategy.

The European Commission signed an APA with AZ in August 2020 to procure 300 million doses of COVID-19 vaccines in stages, with the potential for the EU to obtain a further 100 million doses if needed.^43^ Numerous issues emerged, including missed deadlines and disputes over the number of doses AZ would deliver on time. Consequently, the European Commission accused AZ of breaching its obligations and took legal action against the company in April 2021. The delivery obligations outlined in the EU-AZ APA encountered some difficulties. AZ faced allegations that it was sending more vaccine doses to the UK than to the EU, as the company pointed to its agreement with the UK, which was signed earlier. This prompted the EU to sue AZ for breach of contract due to significantly lower supplies than planned during the first two quarters of the vaccine rollout. The ECA report noted that the Commission’s only options were to take legal action against the vaccine producers by pursuing a case either for failing to make the best reasonable efforts to deliver vaccines or violating the warranty regarding conflicting contracts and failure in delivery.^44^

A settlement followed in September 2021, with AZ agreeing to deliver specified vaccine quantities by designated quarters in 2021 and early 2022. This ended the dispute but impacted regular shipment schedules for EU member states.^45^

The agreement was affected by the fact that AZ’s vaccines were undergoing clinical trials. AZ’s position, the ambiguities regarding pricing and vaccine volumes, delivery, liability waivers, intellectual property (IP) clauses, the safety of AZ’s vaccines, and the lack of transparency in the process are some issues that need to be considered.^46^

One of AZ’s CEOs emphasized that the contract was not a binding commitment but rather a best effort during the dispute. The “best reasonable efforts” clause mentioned in section 1.9 in the EU-AZ’s APA^47^ has faced criticism. Such terms lack a firm commitment to the agreement by the producer. This clause is thought to have been a factor in AZ’s reduced pricing and exemption from responsibility for delays. Schanze mentioned that such clauses are commonly used to soften obligations when future performance is uncertain. However, their inclusion in agreements governing mass vaccine production and delivery has proved problematic, as such vague terms provide manufacturers with more flexibility in meeting supply obligations than firm commitments.^48^ Schanze argues that these clauses were not well suited to the later production stages and concludes with considerations for using alternative contractual frameworks better calibrated to obligate results over uncertain periods.

Heavy redactions initially concealed the pricing details in the EU-AZ’s APA contract. However, a tweet from the Belgian Secretary of State accidentally disclosed these figures, which were later confirmed in the EU and AZ legal proceedings.^49^ In Q1–Q2 2021, Oxford/AstraZeneca priced its vaccine at €1.78 per dose, which was significantly lower than Moderna (€16.82), BioNTech/Pfizer (€^12^), CureVac (€^10^), Johnson & Johnson (€7.14), and Sanofi/GSK (€7.56).^50^ While the WHO advocates for reasonable pricing during emergencies, evidence from the COVID-19 pandemic revealed sharp price escalations across some contracts.^51^

Indemnity clauses played a significant role in the Commission-AZ APAs. However, in the publicly accessible versions of these APAs, most of these clauses are redacted, limiting the ability to conduct further investigations into their specifics.^52^ AstraZeneca’s senior executive team member told Reuters in 2020: “This is a unique situation where we as a company simply cannot take the risk if in … four years the vaccine is showing side effects.”^53^ The Commission and AZ’s APA required each participating Member State to indemnify AZ and related parties, defined as “Indemnified Persons,” from damages and liabilities resulting from vaccine use or administration claims. According to the indemnification clause, the Member States are responsible for compensating individuals and their related persons for various losses resulting from the vaccine, including “death, injuries, illnesses, disabilities, property damage, and business interruption.” This indemnification provides extensive protection for AZ against losses from vaccines supplied under the agreement, regardless of the time or place of vaccination or injury.

The abovementioned clause, therefore, places a significant liability risk on Member States instead of vaccine manufacturers. The broad scope of the clause means AZ is protected at the expense of the Member States and puts financial, legal, reputational, and precedent risks mainly on taxpayers instead of the company.^54^ In 2021, fewer than 20 EU Member States had a vaccine injury compensation program.^55^ Because of the indemnity clause, less affluent Member States could face more significant financial and legal difficulties in managing the costs of vaccine injuries, leading to an uneven risk distribution. Having universal measures such as “The COVAX No-Fault Compensation Program”^56^ or similar (e.g., through an international document such as a “Pandemic Treaty”) could help mitigate the legal risks of the APAs. Although WHO Member States made progress in April 2025 on a consensus on the Pandemic Agreement,^57^ considering the recent challenges the WHO faces, including the USA’s withdrawal from the WHO,^58^ implementing this agreement is likely to be more challenging.

This case study underscores how vague terms and liability waivers can favor producers, potentially constituting anticompetitive abuse of dominance under competition law.

## COVID-19 Vaccines Transparency Cases

The lack of transparency raised important questions about whether the public interest was considered in the APAs between the Commission and pharmaceutical vaccine producers.^59^ This has been highlighted in a number of legal actions which have been brought against the Commission, specifically regarding the Commission’s vaccine strategy during the COVID-19 pandemic. In Joined Cases T-689/21 and T-761/21 (*Auken and Others v. Commission* and *Courtois and Others v. Commission*), members of the European Parliament and private individuals initiated legal action against the European Commission, challenging its failure to provide sufficient public access to the Advance Purchase Agreements (APAs) for COVID-19 vaccines. The Commission justified its redaction of the APAs by citing the need to protect privacy, individuals’ integrity, and the parties’ commercial interests.^60^

The EU General Court held that the European Commission had failed to provide adequate transparency regarding COVID-19 vaccine purchase agreements under Regulation (EC) No 1049/2001 on public access to documents.^61^ The Commission had argued that disclosing the requested information would cause serious and irreparable harm to members of the APA negotiating teams. Nevertheless, the Court annulled the Commission’s refusal to disclose several key categories of information, including the no-conflict-of-interest declarations by negotiating team members and the indemnification provisions of the contracts.^62^ The Commission appealed the ruling, but the judgment is not yet available.^63^

Other countries also have some examples of cases of COVID-19 vaccine transparency, which can be useful for the EU to follow. South Africa’s COVID-19 vaccine procurement contracts case is one of them. On February 22, 2022, the Health Justice Initiative (HJI), a non-profit organization, filed a lawsuit against South Africa’s Minister of Health and the Department of Health’s Information Officer. This followed the Department’s refusal to provide access to COVID-19 vaccine contracts and related agreements in response to a request made under South Africa’s Promotion of Access to Information Act.^64^ The HJI argued that full disclosure of the contracts was vital for public oversight and understanding of one of South Africa’s most significant public procurement processes. The case was heard in the Gauteng High Court, where the HJI contended that the Department’s lack of transparency violated South Africa’s constitution and undermined the public’s right to health information. On August 17, 2023, the Pretoria High Court ruled in favor of the HJI, ordering the full public release of the COVID-19 vaccine contracts and further bolstering transparency and freedom of information in South Africa’s public health governance.^65^

## The Necessity of a New Approach to EU Vaccine Strategy

Because of the lack of established guidelines for direct negotiations and agreements with vaccine producers, the COVID-19 pandemic exposed the EU’s vulnerability in vaccine procurement. In the first purchase agreement between the EU and Pfizer, the price of the vaccine per dose was €15.50. Subsequent agreements increased the price per dose to €19.50. Pfizer and BioNTech renegotiated their contract with the European Commission to deliver COVID-19 vaccines up to 2026.^66^ The prices of these new APAs are thought to be much higher than those of previous ones, although sources remain unconfirmed.^67^

This situation was not so different on the other side of the Atlantic. In an open letter to COVID-19 vaccine manufacturers in July 2023, the Biden administration urged pharmaceutical companies to consider the role of public funding and taxpayers’ money in developing COVID-19 vaccines. In this open letter, manufacturers were also reminded to provide a sufficient supply of updated vaccines with transparent data sharing at reasonable prices for the upcoming new waves of COVID-19.^68^

As the ECA report mentioned,^69^ in order to increase the effectiveness of pandemic preparedness, vaccine procurement and negotiation guidelines must be developed. This study suggests that competition law and the ethics of innovation (among other factors) should be considered when creating such policies. The key characteristics of these guidelines would include clear procurement procedures, defined roles and responsibilities, standard contract formats, performance parameters, key stakeholders, and transparency and accountability mechanisms. These procurement procedures describe how vaccines will be purchased and sourced, including tendering processes, negotiation techniques, legal tools, and criteria for selecting vaccine suppliers.

As Laigle et al. propose, a resilient and harmonious “vaccine ecosystem,” with harmonized policy approaches among member states, could enhance vaccine procurement for future health crises.^70^ The COVID-19 pandemic resulted in a gap between high-income countries (HIC) and low- and middle-income countries (LMIC), which increased the inequities.^71^ The *ex-ante* solidarity-based commitments of vaccine producers and pharmaceutical companies during health emergencies, based on the framework of the pandemic treaty^72^ or a separate international agreement, could help to improve this equity gap. As Towse et al. suggested in their study, *ex-ante* funding commitments from wealthy countries and multinational organizations to pay for the vaccination of low and middle-income countries in a disease outbreak could be a solution.^73^

Another issue that should be considered in the EU vaccine strategy is safeguarding access to vaccines for all through intellectual property (IP) waivers and compulsory licensing. IP waivers are a broader mechanism for temporarily suspending IP rights (including patents, copyrights, and trademarks) during an emergency like a public health crisis. Compulsory licensing is a mechanism in patent law, and it is an authorization that allows governments to grant licenses for patented products without the patent holder’s consent.^74^ Several vaccine manufacturers have strongly resisted the enforcement of vaccine IP waivers and compulsory licensing for COVID-19 vaccines by direct objections or indirect lobbying.^75^

During the COVID-19 pandemic, some countries engaged in vaccine nationalism, hoarding vaccines and limiting exports, which caused supply shortages and other problems. For better managing future pandemics, developing a clear strategy against vaccine nationalism (at the Member State Level) in the EU vaccine strategy is imperative.^76^

In February 2025, the European Commission unveiled “A Competitiveness Compass for the EU: A Roadmap for Growth and Innovation,” its response to the Draghi Report.^77^ The necessity of this roadmap lies in the fact that Europe, even with a vast single market and social economy, is falling behind in tech leadership and is aiming to fill this gap by focusing on “simplification,” “deregulation,” and “increasing competitiveness.” The roadmap focuses on “growth, innovation and sustainability” in a five-year landscape. The next steps will be aligning the EU policies through “the Competitiveness Coordination Tool” and establishing a “European Competitiveness Fund,” which is also a big part of this roadmap.^78^

In light of the experience of the COVID-19 APAs and their limited transparency, pricing practices, and indemnification, a set of principles can be set out that can underpin future APAs and the design of future vaccine strategies.

## The FACER Framework

This study proposes **the FACER Framework** to address the shortcomings exposed in the European Union’s procurement of COVID-19 vaccines and to increase its preparedness for future public health emergencies. The framework comprises five pillars: Fairness, Accountability, Competition Law, Ethics of Innovation, and Resilience. The FACER Framework can be used as a theoretical framework rooted in legal and ethical considerations and offers a concise and adaptable approach to rethinking vaccine strategies.

The FACER Framework aligns with the TFEU’s enforcement mechanisms and the TEU and Charter of Fundamental Rights of the European Union (CFR) values while embedding a strong ethical core to explain and recommend reforming the EU vaccine strategy. In doing so, it addresses the concerns raised in negotiating APAs and vaccine procurement processes during the COVID-19 crisis, including the accountability deficits and market distortions that undermined the EU’s response to the pandemic. By using competition law and ethics of innovation as accountability measures, FACER provides a pathway to strengthen the EU’s resilience in confronting future health challenges.

### Pillar 1: Fairness through Equitable Procurement

Fairness is a core principle of the FACER framework, ensuring that vaccine procurement and distribution are guided by fairness as an ethical principle, and the EU should consider fairness when designing vaccine strategies. Fairness in public health focuses on ensuring everyone can access vaccines in a health crisis, regardless of social and economic factors or geopolitical background. Fairness (closely connected to equity and justice in this context) involves transparent procurement processes, fair pricing, and global solidarity, as seen in efforts like COVAX. This pillar emphasizes that equitable distribution of both the benefits and risks of vaccines between pharmaceutical companies and the EU is important, and a fair contracting model that includes indemnification clauses should consider this fact. As Dai et al. discussed, there must be a trade-off between fairness and efficiency in designing vaccine strategies.^79^

During the pandemic, serious concerns were raised by Member States, EU authorities, and the general public about how the APAs lacked transparency and potentially favored big pharmaceutical companies over the public interest. This study highlighted broad liability waivers in APAs, such as those with AstraZeneca, which shifted risks to member states and taxpayers, potentially undermining Fairness.

Although the timely procurement of vaccines for Member States is an important priority, as mentioned in the EU vaccine strategy, it is also essential that Fairness extends beyond the EU (based on global solidarity, which aims to share vaccines with lower-income countries).^80^

### Pillar 2: Accountability and Transparency in Vaccine Procurement

The second pillar is focused on pharmaceutical companies’ accountability regarding vaccines and transparency in the central vaccine procurement process.

Using communication tools such as Short Message Service (SMS)^81^ as the primary method for negotiating to purchase more than 900 million doses was one of the Commission’s unprecedented strategies during the pandemic. The Commission’s publication of a heavily-redacted version of its APA with AZ, and the casual communication tools used during vaccine negotiations between the Commission and Pfizer’s CEO highlight how a lack of transparency and proper oversight can erode the public’s trust.^82^ Insisting on releasing essential details (such as liability terms) would open the door to examining potentially unfair market practices and honoring the public’s rightful interest in vaccines in part funded by their taxes. This echoes Organisation for Economic Co-operation and Development (OECD) research which emphasizes transparency as a bedrock of trust in health policy.^83^

The successful transparency lawsuits around COVID-19 vaccine contracts in EU courts and South Africa could serve as an influential legal precedent for making vaccine procurement and negotiation more transparent and holding the pharmaceutical sector more accountable in the future.

### Pillar 3: Competition Law in Vaccine Procurement

Competition law ensures fair competition and maintains a level playing field in the market.^84^

The European Commission, national competition authorities, and courts monitor anticompetitive practices in the pharmaceutical sector and enforce Article 101 of the TFEU (anticompetitive agreements between pharmaceutical undertakings) and Article 102 (abuse of a dominant position).^85^ In the pharmaceutical sector, EU competition law aims to prevent anticompetitive practices that harm patients’ access to affordable and innovative medicine. Before the pandemic, the Commission investigated and fined several pharmaceutical companies for anticompetitive agreements and practices such as pay-for-delay settlements (when originator companies pay generic pharmaceuticals to delay market entry) and unfair and excessive pricing.^86^ However, the COVID-19 pandemic exposed competition law vulnerabilities, with a few vaccine producers dominating the market. The vaccine market is inelastic, and the lack of alternatives, especially in the first few months after the vaccine development, resulted in the powerful position of vaccine producers on the market and led to pricing variations between countries without cost justifications. Anticompetitive practices, such as excessive and discriminatory pricing from vaccine producers (for example, implementing a tiered pricing strategy by Pfizer and BioNTech during the COVID-19 pandemic), could distort the competition in the market during a crisis.^87^

The EU’s vaccine strategy can be directly threatened by anticompetitive agreements such as price-fixing. Those measures go against the EU’s principle of solidarity and could worsen health inequalities.^88^ Lack of trust in the vaccination process and public health authorities caused by the anticompetitive practices of pharmaceutical companies can result in more vaccine hesitancy. The unique and unprecedented nature of the COVID-19 crisis, high demands for COVID-19 vaccines, a lack of clear and transparent policies regarding vaccine procurement negotiations, and the limited number of vaccine producers gave them colossal bargaining power in negotiations with EU officials. These negotiations may exhibit abuse of bargaining power, which can fall under the concept of abuse of dominance in competition law.^89^ Although merely having bargaining power is not against the law, as parties may pursue favorable outcomes using legal means during negotiations, exploiting this power is detrimental to the market process.

Most of the concerns in the pharmaceutical sector are related to antitrust. However, mergers in this sector can pose significant risks to market dynamics, particularly during a pandemic. While vertical integration through a merger may yield efficiencies, it sometimes increases barriers to entry, stifling competition and innovation. Such trends, if unchecked, could increase vulnerabilities in future crises by limiting the diversity of vaccine or drug suppliers, potentially leading to supply shortages and price distortions. This concentration raises concerns under Article 101 TFEU and the EU Merger Regulation (Council Regulation (EC) No 139/2004), as strategic alliances or mergers might facilitate anticompetitive coordination, undermining equitable access.

The Commission should closely monitor mergers and acquisitions in vaccine development, R&D, and manufacturing as part of an effective vaccine strategy.^90^ A key area of focus for the Commission in the biopharmaceutical sector (including vaccine markets) is “killer acquisitions,” where larger companies acquire smaller, innovative firms, which may eliminate future competition. A study published by the Commission in November 2024 showed that market consolidation, which may occur through various means, could positionally reduce research expenditure and patent output.^91^ Considering the strategic importance of vaccines, especially highlighted by the COVID-19 pandemic, the FACER framework recommends that the EU vaccine strategy integrate robust competition law considerations. As the Draghi Report on EU competitiveness emphasizes,^92^ the need for a forward-looking approach to competition policy, particularly in pharmaceutical vaccine markets, is imperative to ensure dynamic efficiency and productivity. A well-defined vaccine strategy should embed potential competition concerns arising from collaborations, abuse of dominance, or mergers to guarantee a diverse, competitive, and resilient landscape for vaccine research, development, and manufacturing within the EU.

### Pillar 4: Ethics of Innovation in Vaccine Development

Ethics of Innovation reframes vaccine development as a package of benefits and moral responsibility,^93^ asserting that pursuing new solutions must place fairness above exclusionary practices. As McMahon argues, patents give holders specific control and “private governance” over new medical inventions.^94^ Patent holders may impact equitable access to pandemic-related products, including vaccines. Abusing IP rights while in a dominant position during a pandemic could negatively impact global health equity.^95^ Broad financial indemnity clauses, zero or less flexibility over IP waivers, and vague clauses regarding what ought to be key terms of APAs (such as vaccine delivery time and quality expectations) could be signs of abusing the IP-conferred powers.

Because of the use of taxpayer money and governmental funding in vaccine development, there is a strong case for establishing legal mechanisms that impose a special responsibility on IP holders to share technology during public health crises. Such measures could include IP law flexibilities, such as IP waivers, compulsory licensing, combating pay-for-delay arrangements, product hopping of pharmaceuticals, and other legal tools to ensure equitable access to essential medical innovations.^96^ Ethical considerations inform this approach. However, it is the law that should provide enforceable obligations on rights holders in the context of global health emergencies.

Geiger and McMahon argue that global mechanisms for equitable access to COVID-19 vaccines, such as “COVAX,” the WHO’s “Accelerator (ACT-A)” initiative, the WHO’s “COVID-19 Technology Access Pool (C-TAP),” proposed World Trade Organization (WTO) technology transfer hubs, and the “TRIPS intellectual property waiver,” had complementary features.^97^ They suggest that greater coordination between the WHO, the WTO, and supporting states is necessary to address shortcomings in global crisis management and enhance equitable access to vaccines. Despite its shortcomings, the Commission’s new proposal to establish a “compulsory licensing scheme”^98^ for pharmaceuticals and vaccines during a crisis is a significant step towards providing European access to medicine.^99^ However, Pharmaceutical companies strongly opposed a new scheme, claiming it would weaken innovation in the sector.^100^

The reaction of the pharmaceutical industry to public health emergencies such as HIV and COVID-19 showed us how the opposition of this industry to IP waivers and compulsory licensing has often reinforced market dominance and deepened inequalities.^101^ While competition law tackles monopolistic tendencies, this perspective argues that research and development supported by public funds are ethically obligated to disseminate knowledge and promote accessibility, perhaps via mechanisms like the European Union’s compulsory licensing framework.^102^

### Pillar 5: Resilience Against Systemic Distortions

The Draghi report on “The Future of European Competitiveness” emphasized building resilience in the EU economy through the development of a system sturdy enough to endure crises, mainly through stronger supply chains in critical sectors (including the pharmaceutical industry).^103^ The report does not directly address the EU vaccine strategy (although it discusses explicitly the pharmaceutical sector and vaccine producers). However, Draghi’s recommendations (including enhancing industrial coordination and innovation) indirectly support a more robust vaccine strategy, several of which have been reflected in the European Commission’s 2025 Competitiveness Compass, particularly in areas related to industrial coordination, innovation, and resilience planning.^104^

Many problems regarding COVID-19 vaccines were directly or indirectly related to the fifth pillar of the FACER Framework. Delays in vaccine deliveries were partly due to reliance on non-EU manufacturers, particularly for mRNA vaccines. Supply chain distribution was exacerbated by global competition and export controls, specifically in early 2021, and coordination issues among Member States highlighted a lack of resilience in the EU’s health infrastructure.

## Conclusions

This research reviewed the EU vaccine strategy, specifically focusing on the advanced purchase agreements (APAs) during the COVID-19 pandemic. The analysis identified some accomplishments, such as establishing joint procurement to increase bargaining power and promote intra-European solidarity, and some concerns, especially regarding accountability mechanisms, transparency, competition law, and ethics of innovations.

Embedding competition law and the ethics of innovation into future vaccine strategies, as this study proposed through the FACER Framework, offers a transformative path forward. Fairness, Accountability, Competition law, Ethics of innovation, and Resilience are the five pillars of the FACER framework, and they address and propose solutions to the deficiencies in the EU vaccine strategy. Fairness ensures equitable dose allocation and counters vaccine nationalism, while Accountability mandates transparency and oversight to rebuild public trust. Competition Law curbs anticompetitive practices and agreements like excessive pricing or cartels, which could also happen because of the crisis. Ethics of Innovation reframes vaccine development as a moral duty to share publicly funded knowledge. Resilience fortifies the system against disruptions through forward-looking planning and economic and industrial strength for future public health crises. The AstraZeneca APA exemplifies how these principles could have mitigated vague terms, liability shifts, and dominance abuses, ensuring a more just outcome. The FACER Framework provides a thoughtful plan for weaving competition law and ethical principles into the EU’s vaccine strategy. Adopting FACER could equip the EU to face future health crises with sharp legal insight and moral clarity, possibly offering a model for global health policy to follow.
